# A qualitative assessment of mental health literacy and help-seeking behaviors among male college students

**DOI:** 10.1007/s44192-022-00028-9

**Published:** 2022-12-29

**Authors:** Rita DeBate, Amy Gatto, Gregor Rafal, Jennifer Bleck

**Affiliations:** 1 College of Public Health, University of South Florida, 13201 Bruce B. Downs Blvd, Tampa, FL 33612 USA; 2 Colorado Department of Public Health and Environment, 4300 Cherry Creek Dr. S, Denver, CO 80246 USA

**Keywords:** Men, Mental health, College health, Mental health literacy, Help-seeking behaviors, Qualitative

## Abstract

Although the number of students receiving care from college counseling centers has increased, engaging male college students to seek help presents a unique challenge. This qualitative study explored mental health literacy and help-seeking behaviors among undergraduate college men. Semi-structured interviews (n = 26) based on three vignettes (anxiety, depression, stress) were employed to assess mental health literacy. Analysis revealed three general themes and associated sub-themes: (a) knowledge of signs and symptoms (physiological, behavioral, and emotional); (b) recommended help-seeking behaviors (do nothing, self-care, seek help); and (c) barriers to help-seeking (social stigma, self-stigma, masculinity). Findings present a triadic interplay between the person, help-seeking behavior, and environment. Future research should explore this dynamic relationship to inform interventions aimed at improving college male mental health help-seeking behavior.

## Introduction

Due to various unique stressors, emerging adulthood, the age group for most college students, is a critical period of the life course [1]. Poor mental well-being among young adults is an important public health issue as mental health symptoms have doubled among college students [2]. Prevalence data (2013–2021) from a large national study of students from 373 colleges/universities revealed worsening trends in flourishing (− 32.5% change), depression (+ 134.6% change), anxiety (+ 109.5% change), eating disorders (+ 95.6% change), suicidal ideation (+ 64% change), and non-suicidal self-injury (+ 45% change) [3]. Addressing mental well-being among college students is particularly important as poor mental health has been identified to be associated with binge drinking, physical inactivity, poor diet, high stress, anxiety, loneliness, poor body image, interpersonal issues, co-occurring mental health concerns, and discrimination [4, 5]. If left untreated, mental health disorders have been indicated as a risk factor for decreased academic productivity and a two-fold increased risk of dropping out without graduation [6–8]. As such, the American College Health Association identified several objectives to improve college student mental health such as reducing attempts as suicide, reducing disordered eating, and increasing diagnoses of mental health issues [9]. 

### Mental health help-seeking behaviors among college males

While data point towards an increase in the number of students receiving care from college counseling centers [10], engaging college men to seek help is a unique challenge [11, 12].

Data from the 2022 American College Health Association-National College Health Assessment reveal that there was little difference in the percentage of male and female college students who reported experiencing moderate/severe psychological distress, loneliness, or a positive screening for suicidal ideation. However, only 36% of male undergraduate college students reporting ever receiving psychological or mental health services as compared to 54% of females [13].

Alternatively, male college students are more likely to engage in unhealthy coping mechanisms in place of seeking care [4, 12, 14]. Chuick and colleagues [15] noted a cyclical relationship between symptoms of a mental health concern, external consequences, and coping mechanisms in that males who participated in negative coping strategies further exacerbated their mental health symptoms [15]. Interestingly, although men have lower reported rates of depression [16], males have also been observed with a higher suicide rate (12.6 per 100,000) as compared with females (5.4 per 100,000) [17].

### Mental health literacy among college males

Mental health literacy (MHL), defined as “knowledge and beliefs about mental disorders which aid their recognition, management, or prevention,” [18] has been identified as an avenue to increase students mental well-being and help-seeking behaviors [19, 20]. Despite multiple studies assessing mental health literacy among young adults and college students, few have focused solely on males residing in the U.S. Of those that have examined MHL among college students using survey methodology, studies contained low sample sizes for male participants [14, 21–26], focused on a single mental health issue (e.g., depression) [27–31], or were conducted outside of the United States [12, 14, 22–24, 26, 29].

Nonetheless, current research suggests low MLH among males as compared to their female counterparts [11, 32–36]. Aspects of MHL identified as barriers to male mental health help-seeking behaviors include: (a) male gender norms, including masculinity [12, 15, 27, 37–41]; (b) stigma [11, 12, 27, 40, 42–45]; and (c) attitudes regarding mental health [12, 42, 45]. Additional factors revealed in the current literature include discounting the severity of their mental health concern or feeling as though professional treatment was not necessary; (b) confidentiality, lack of trust, and fear; (c) self-reliance and seeking help from other resources; and (d) accessibility, time, and cost [6, 46–51]. For example, a cross-sectional study assessing depression-specific help seeking behaviors among 313 college males attending a Midwestern university, revealed relationships between gender role conflict, self-stigma, and help-seeking behaviors [27]. More specifically, self-stigma predicted a decreased willingness to engage in formal and informal (e.g., family, friends) help-seeking while gender role conflict was found to be associated with decreased willingness to engage in informal help-seeking behaviors and increased avoidant behaviors [27]. A meta synthesis of studies published from 2010 to 2013 which focused on men’s perspectives of mental health (n = 26), revealed the following themes regarding mental health literacy (a) pressure to subscribe to dominant masculine ideals; (b) inability to recognize and articulate mental health issues; and (c) a desire to manage the issue as opposed to help-seeking [30]. Nonetheless, due to limitations in participant sampling (e.g., middle class, educated male populations diagnosed with depression) the authors recommend future research that explores the perspective of men who are not sampled solely on their experience of having diagnosed or self-reported depression [30]. Similarly, the authors of a scoping review of male mental health published between 2010 and 2020 (n = 21) note that although stigma was a common experience among male participants, the literature regarding male mental health literacy remains undeveloped [40]. To that end, the authors emphasize the need for qualitative research exploring the nature and mechanisms regarding stigma and mental health literacy among diverse groups of males across the life course [40].

Addressing above-described recommendations for future male mental health research, the purpose of the current qualitative study was to gain a better understanding of the multiple facets of mental health literacy among emerging adult males attending a U.S. based university. As mental health literacy is embedded in various cultural contexts, qualitative methodology was employed to further explore dimensions of mental health literacy within the context in which they occur [52].

## Materials and methods

### Participants

This study utilized a purposive sample of undergraduate males at a large southern research university in the United States. Inclusion criteria for the study included: (a) ≥ 18 years old; (b) self-identify as male; and (c) currently enrolled in an undergraduate degree. Data collection occurred from June to September 2017. The procedures used in this study adhere to the tenets of the Declaration of Helsinki.

### Recruitment

Participants were recruited through e-mail and university-specific social media which are moderated by university administrators. In addition to a description of the study, recruitment materials included a link to an online survey to assess if interested males met inclusion criteria. If inclusion criteria were met, additional contact information was gathered to enable researchers to schedule 30- to 60-min in-person interviews.

Prior to each interview, participants were asked to read and sign an informed consent form; the procedures and purpose were explained by the moderator; and participants were given an opportunity to ask questions. All interviews were audio-taped and later transcribed verbatim, excluding identifying information, by an experienced transcriptionist. Those who participated in the study were compensated with a $10 University Dining Services meal voucher. Research integrity and compliance approval was granted through the University’s Institutional Review Board prior to study implementation.

### Conceptual framework

An expanded definition of mental health literacy, offered by Jorm [53] served as the conceptual framework for this study. Originally defined as “knowledge and beliefs about mental disorders which aid in their recognition, management, or prevention” [54], Jorm indicated that although knowledge and beliefs regarding mental health are necessary for prevention, recognition, and management of mental health issues, they are not sufficient for improving mental health as the knowledge must be linked to mental health help-seeking behaviors to assist self and other [53]. Translation of this expanded definition of mental health literacy informed the following research questions: (a) what are college male students’ perceptions regarding mental health (e.g., knowledge, beliefs, attitudes, susceptibility, severity); and (b) what are college male students’ perceptions regarding mental health help-seeking behaviors (e.g., for self, friend, other).

### Measures and procedures

Although, there has been disagreement regarding the proper way to conceptualize and study mental health literacy [55, 56], one method that has been used widely among young adults is the use of vignettes illustrating the mental health symptoms and asking the participant to identify the disorder [14, 21, 22, 24–26, 29, 57].

Interviews utilized three vignettes representing a male college student presenting with anxiety, depression, or stress. Guided by diagnostic criteria according to the Diagnostic and Statistical Manual of Mental Disorders-5 [58] vignettes were developed by a member of the research team who is a licensed psychologist experienced in working with university students. Three additional licensed psychologists reviewed each vignette for face (i.e., the extent to which the vignettes appear to be assessing signs and symptoms of generalized anxiety disorder, depression, stress) and content validity (i.e., extent to which the vignettes contain samples of signs and symptoms of generalized anxiety disorder, depression, stress) with respect to a college student population [59]. Table 1 includes the vignettes and accompanying text representing DSM-5 [58] mental health disorders.

**Table 1:** Study vignettes

Disorder	Vignette
Anxiety	John is 20 years old, works part-time, and is currently enrolled in classes at USF. He complains of dizziness, heart palpitations, ringing ears, trembling, and sweating palms. Additional periodic symptoms include a sore throat, cough, or dry mouth and throat. Periods of extreme muscle tension, along with feelings of being "wound up" or "edgy" are also present. These symptoms often interfere with his concentration and have been present more often than not for about the past 2 years. John constantly worries about failing his classes, completing projects at work in a timely manner, being able to please his parents, and paying bills. He keeps a low profile at work, and also avoids going out with friends, meeting new people, and exercising at the gym
Depression	Marcus is 21 years old. He has been feeling unusually sad and miserable for the last few weeks. Even though he is tired all the time, he has trouble sleeping nearly every night. Marcus doesn't feel like eating and has lost weight. He can't keep his mind on his schoolwork and puts off making any decisions. Even day-to-day tasks seem too much for him. This has come to the attention of Marcus’s advisor who is concerned about his poor academic performance. Marcus feels he will never be happy again and believes his family would be better off without him. Marcus has been so desperate; he has been thinking of ways to end his life
Stress	Steve is 20 years old. For the past week, he has been experiencing sleep difficulties, and digestive upset. He recently changed his major and his part-time job responsibilities have increased. Nevertheless, Steve has not missed any of his classes, has consistently arrived on-time at work, completes his school-related assignments, manages to attend his intramural sport games, and continues to occasionally socialize on weekends. Additionally, this past week he has been feeling irritable and pressured, and experiencing intermittent headaches

The vignettes comprised of text and accompanying photo were presented to each participant. Photos were included for the purposes of increasing identification of each vignette to a typical male college student attending the university-specific setting (e.g., photo of a college male (varying in race/ethnicity) wearing university-specific logo with the university-specific setting).

Interviews were conducted in a semi-structured manner; the same series of questions were asked for each of the three vignettes and probing for additional information was left to the discretion of the interviewer. Interview questions were arranged in a funneling pattern according to protocol outlined by Hawe and colleagues [60]. Key questions included: (a) Given what I just described about *name from vignette*, what do you think *name from vignette* is experiencing; (b) Explain why you answered that way; (c) Describe how *name from vignette* can best be helped; (d) Where do you think he could get more information about this concern; (e) Imagine *name from vignette* is someone you’ve known for a long time and you know him well. You want to help him. What do you think is appropriate to do; (f) Have you or any of your friends ever experienced this? What did you do/tell them to do; and (g) If you were *name from vignette* what would you do? As a means to increase respondent cooperation and data quality regarding this “sensitive” topic, interviews were conducted by one male researcher as it has been noted that interviewer characteristics such as sex can affect respondent-interviewer rapport; thus matching interviewers with respondents on that characteristic as to increase comfort, rapport, and trust [61].

At the end of each interview, participants were asked to complete a paper–pencil questionnaire to assess perceived helpfulness of various mental health resources [62]. Sources of mental health help included parents, friends, mental health specialists, religious leaders, academic personnel, and telephone/text help lines, among others. Answer categories presented on a five-point Likert type scale ranging from very helpful to very unhelpful. An “I don’t know” answer category was also included. A second questionnaire captured participant’s demographic information and included race, ethnicity, age, and college of study.

### Data analysis

Throughout the data collection process interview audio files were transcribed and reviewed for accuracy by the study team. Three independent coders who were members of the research team initially hand-coded the interview transcripts. Individual codes within the code families were garnered through inductive investigation of the data. Members of the research team were responsible for determining which codes presented in a code family. The research team then met and discussed individual codes until consensus was reached. This process was repeated to reduce the number of individual codes such that themes might present more readily. To ensure inter-rater reliability at least three research team members had to completely agree on the code for each quotation. Coding of the study transcripts was performed using Atlas.Ti v6.2 where axial coding was then performed to identify overarching themes and sub-themes.

Descriptive statistics were run to examine the frequency of all quantitative survey responses using SPSS 24. For the perceived helpfulness scale, because of the small sample size, the answer categories for the 5-point Likert scales were collapsed into three categories, helpful, neutral, and not helpful. Prior use questions were scored as dichotomous yes/no variables.

## Results

Twenty six in-person interviews were conducted reaching data saturation (i.e. the ability to no longer gain any new information [63]). Table 2 depicts demographic and behavioral characteristics of the sample. Among participants, the majority were non-Hispanic (76.9%), white (53.8%), and non-international students (92.3%). Participants’ year in college ranged from first to 15 + years, but most students were either in their second (38.5%) or third (38.5%) year. The college of study of participants was diverse and included art, arts and science, behavioral science, business, engineering, and public health. The mean age of the sample was 20.6 years old. The student health center was reported to be the only campus health resource used by over half of the sample (57.7%). With regard to correctly identifying the mental health concern for each of the vignettes, 14 participants (53.8%) correctly identified anxiety, 25 (96.2%) correctly identified depression, and 19 (73.1%) correctly identified stress.

**Table 2:** Demographic and behavioral characteristics of Male Participants (n = 26)

	n (%)	
*Ethnicity*		
Non-Hispanic	20 (76.9)	
Hispanic	6 (23.1)	
*Race*		
African American	2 (7.7)	
Asian	10 (38.5)	
White	14 (53.8)	
*International Student*		
No	24 (92.3)	
Yes	2 (7.7)	
*Living Status*		
Off-campus	23 (88.5)	
On-campus	3 (11.5)	
*Year in School*		
1st year	2 (7.7)	
2nd year	10 (38.5)	
3rd year	10 (38.5)	
4th year	3 (11.5)	
5th + year	1 (3.8)	
*College of Study*		
Arts	1 (3.8)	
Arts and Science	9 (34.6)	
Behavioral and Community Science	3 (11.5)	
Business	4 (15.4)	
Engineering	8 (30.8)	
Public Health	1 (3.8)	

Age	Mean	SD
	20.64	3.04

Prior use of on-campus resources	Yes, n (%)	No, n (%)
Counseling Center	10 (38.5)	16 (61.5)
Psychological Services Center	1 (3.8)	25 (96.2)
Student Health Services	15 (57.7)	11 (42.3)
Student Outreach and Support	1 (3.8)	25 (96.2)
Wellness Center	8 (30.8)	18 (69.2)

Analysis of qualitative data yielded three general themes: (1) knowledge of signs and symptoms; (2) recommended help-seeking behaviors, and (3) barriers to mental health help-seeking behaviors. Table 3 depicts a summary of general themes and associated sub-themes. Themes and sub-themes are described in detail within the following sections.

**Table 3:** MHL themes and subthemes among male college student participants

Knowledge of signs & symptoms	Recommended help-seeking behaviors	Barriers to help-seeking behaviors
Physiological	Interpersonal (unknown male)	Social stigma
	• Seek mental health services	• Family and friends’ perceptions
Behavioral		• Fear of losing friends
	Interpersonal (male friend)	• Outcast
Emotional	• Do nothing	
• Suicidal ideation	• Talk with friend	Self-stigma
	• Seek medical health services	• Control
		• Pride
	Intrapersonal (self)	
	Anxiety	Masculinity
	• Do nothing	• Gender norms
	• Self-care	
	Depression	
	• Do Nothing	
	• Seek mental health services	
	Stress	
	• Self-care	

### Theme one: knowledge of signs and symptoms

Analyses revealed that for all three mental health issues presented via the vignettes, participants demonstrated knowledge regarding identification and descriptions of signs and symptoms of poor mental well-being. Three sub-themes emerged in that knowledge of signs and symptoms were described by three types including: (a) physiological; (b) behavioral; and (c) emotional. Moreover, comparable sub-themes were revealed when comparing participants who correctly identified the mental health issue portrayed in the vignette to those who did not correctly identify the presenting mental health issue. That is to say, the majority of male participants identified and described physiological, behavioral, and emotions signs and symptoms indicating “the person in the vignette is experiencing something”, but as presented previously, not all participants were able to identify the issues as either anxiety, depression, or stress. Furthermore, most participants who did not describe vignettes as experiences of poor mental health described the signs and symptoms displayed in the vignette as “normal” to the college experience.

*Signs and symptoms: physiological* For all three vignettes, physiological signs and symptoms were identified recurrently as compared to behavioral and/or emotional. Physiological indicators included heart palpitations, sweating palms, trembling, dizziness, fatigue, loss of appetite, and weight loss. Examples of participant quotes representative of this subtheme include the following:

*[Anxiety] “The physical symptom, dizziness, heart palpitations, sweating palms, trembling, sounds like an indication of anxiety … it sounds like his physical symptoms match his mental state.”*

*[Depression] “He's losing weight, he does not feel like eating, he's constantly tired … he just lacks the energy.”*

*[Stress) “Major stress. … his body is starting to show the physical signs of being overwhelmed and overworked. Upset stomach … and the headaches too.”*

*Signs and symptoms: behavioral* The second sub-theme emerged from the analyses revealed the expression of behavioral signs and symptoms of poor mental well-being. Behavioral signs and symptoms included lack of self-care, trouble sleeping, lack of focus, avoidance, and social isolation. Examples of participant quotes representative of this subtheme include the following:

*[Anxiety] “He talks about wanting to be isolated and introverted, staying low profile at work, and he doesn't want to go out with his friends and meet new people.”*

*[Depression] “…constantly wanting to sleep, but when he does try to sleep, he can't.”*

*[Stress) “It seems like he's got a lot on his mind, which I guess is causing him not to be able to sleep well”*

*Signs and symptoms: Emotional*The third sub-theme emerging from the analyses revealed emotional signs and symptoms of poor mental health. Although emerging as a sub-theme, emotional signs and symptoms were expressed the least out of the three sub-themes. Emotional signs and symptoms identified among the three vignettes included worry, irritation, on edge, and pressure. Examples of participant quotes representative of this subtheme include the following:

*[Anxiety] “‘Wound up, edgy.’ I guess he has anxiety all the time…he's just constantly worried all the time.”*

*[Depression] “He's unusually sad and miserable”*

*[Stress] “Feeling irritable and pressured”; “It seems like he's got a lot on his mind…and he says he's feeling pressured”*

Additionally, although not a sub-theme per-se, specific to the depression vignette, participants identified and described suicidal ideation was an indication of depression. Examples of participant quotes representative of this subtheme include the following:

*“Thinking of ways to end his life. That would be the big highlight right there.”*

*“Suicidal thoughts and tendencies, that's kind of one of the big signs I guess.”*

*“You start thinking about ways to end your life … It's definitely symptoms of that [depression].”*

### Theme two: recommended help-seeking behaviors

The second general theme that emerged from analysis pertains to the discrepancy between what the participant would recommend for a male stranger exhibiting signs and symptoms of poor mental health versus what he would advise for a male friend versus what he would advise for himself. Participants advocated distinct help-seeking strategies contingent upon their association with the person in question. Accordingly, this theme comprised of two sub-themes including: (a) Intrapersonal (Self); and (b) Interpersonal (Male Friend, Unknown Male).

#### Recommended help-seeking behaviors for self

With reference to recommended intrapersonal help-seeking behaviors, participants conveyed three main strategies for addressing presenting mental health issues in the vignettes. Although differences were observed depending on the presenting mental health issue, recommended strategies included: (a) do nothing; (b) engage in self-care; (c) seek mental health services. When presented with the anxiety and/or the depression vignette, participants expressed that they would “do nothing.” The self-recommendation to “do nothing” was supported by the belief that described signs and symptoms were not severe and they believed they would be able to work through it on their own. Frequent terms to express this belief included “pushing through” and “waiting it out.” Examples of participant quotes representative of this subtheme include the following:

*“Just kept working through it … Things are going to get tough in life, and I just kind of ended up dealing with it. Pushed through. Grass is always greener on the other side.”*

*“I would just wait until I had some free time or if until things got kind of serious.”*

Engaging in self-care was articulated when presented with the anxiety and/or stress vignette. Self-care practices suggested by participants covered a broad range of items including wellness activities such as breathing exercises, physical exercise, relaxation methods, and organizing one’s schedule. Examples of participant quotes representative of this subtheme include the following:

*“…reset my diet, my sleep schedule, you know, spend more time just meditating and just thinking about what I can do to positively benefit my life.”*

*“And I would make sure that I've not overworked myself. That I had time … an hour or an hour and a half to myself just relaxing.”*

*“I would definitely start cutting back on some activities to just try to remove some of that load from my life.”*

Seeking mental health services was only expressed when presented with the depression vignette. As depicted in Table 4, participants indicated the usefulness of different mental health services included peers, family members, and healthcare providers. Examples of participant quotes representative of this subtheme include the following:

*“That's the way it feels, but if you actually put in the time to go to counseling and try to help yourself, then you can get out of it.”*

*“I would probably try to seek help in the wellness center or a therapist’s office or something.”*

**Table 4:** Perceived usefulness of resources for mental health concerns (n = 26)

	Very/Helpful n (%)	Neutral n (%)	Very/Unhelpful n (%)
Friend from university	25 (96.2)	1 (3.8)	–
Counselor	23 (92.0)	2 (8.0)	–
Friend outside university	22 (84.6)	4 (15.4)	–
Mental health professional at Counseling Center	22 (91.7)	1 (4.2)	1 (4.2)
Psychologist	22 (95.7)	1 (4.3)	–
General practitioner (GP)	21 (84.0)	3 (12.0)	1 (4.0)
Psychiatrist	21 (91.3)	2 (8.7)	–
Parents	19 (73.1)	3 (11.5)	4 (15.4)
Partner/spouse	18 (90.0)	1 (5.0)	1 (5.0)
Wellness coach	18 (94.7)	1 (5.3)	–
Physician at Student Health Services	17 (81.0)	4 (19.0)	–
Organization to help people deal with their problems	16 (72.7)	2 (9.1)	4 (18.2)
Other relative	16 (64.0)	4 (16.0)	5 (20.0)
Resident Advisor (RA)	13 (56.5)	6 (26.1)	4 (17.4)
Academic advisor	12 (50.0)	9 (37.5)	3 (12.5)
University professor	12 (48.0)	11 (44.0)	2 (8.0)
Telephone counseling service	8 (40.0)	7 (35.0)	5 (25.0)
Clergy/religious priest	7 (36.8)	6 (31.6)	6 (31.6)
Web-based counseling service	7 (36.8)	5 (26.3)	7 (36.8)
Spiritual leader	5 (27.8)	8 (44.4)	5 (27.8)
Not approach anyone for help and deal with the problem alone	3 (11.5)	3 (11.5)	20 (76.9)

#### Recommended help-seeking behaviors for a male friend

The interpersonal sub-theme encompasses recommendations participants would present to their male friends and other males. With regard to recommendations to their male friends, in general across mental health concerns, participants conveyed that they would either “do nothing” or talk to their friend about signs and symptoms, and/or suggest that the friend seek assistance from healthcare or a mental health provider. When presented with the depression and anxiety vignettes, most participants expressed that they would not provide any recommendations for help-seeking to a friend. When data were compared by those who correctly identified the depression and anxiety vignettes to those who did not, the recommendation remained consistent. The majority of participants expressed that due to a stated lack of qualifications, they did not believe they were in the position to intervene or were unsure of how to appropriately handle such a situation. Since the depression vignette indicated suicidal ideation, many participants conveyed concern in that if they said the wrong thing it would make the situation worse. Examples of participant quotes representative of this subtheme include the following:

*“I personally believe that I shouldn't be telling people what to do.”*

*“It's his life so I feel like it's his responsibility to improve his wellbeing.”*

*“I don't know what I can do personally, since I wouldn't really know how to deal with this.”*

*“If I give him some suggestion and he takes it the other way, it will just be bad for him.”*

Irrespective of mental health issues presented in the three vignettes, participants expressed that they would talk with their friend about signs and symptoms. For example, participants expressed that their friend may have been suppressing signs and symptoms of the mental health concern, and by addressing these with their friend, could provide useful. Examples of participant quotes representative of this subtheme include the following:

*“For me to do as a friend, would be to listen to how he's feeling.”*

*“Just tell him straight up, "Hey, you've been saying you have all these symptoms going on. I think you may have a bit of high anxiety.”*

*“Maybe talking with him … and working through it, and trying to make him feel a little better.”*

Lastly, when talking with their friend, most participants expressed that they may also recommend that the friend seek assistance from a medical professional as a way to address physiological issues. Males recommending mental health services described this recommendation as a measure for suicide ideation. Examples of participant quotes representative of this subtheme include the following:

*“I'd talk to him and say, You know. First thing, seek medical professional help. The doctors.”*

*“I think the first thing would definitely be, make sure he gets to a doctor, gets checked up, make sure that everything physically is fine.”*

*“If he's been talking about ending his life he should see a counselor of some sort.”*

#### Recommended help-seeking behaviors: unknown male

Across all three mental health concerns, when the person described was an unfamiliar male, participants conveyed that the male should seek assistance from a mental health provider. This recommendation held true whether the participant correctly or incorrectly identified the mental health issue presented in the vignette. Examples of participant quotes representative of this subtheme include the following:

*“I think John could use maybe some counseling services.”*

*“He should go visit a psychiatrist or something so that they can go through what's happening to his day-to-day life”*

*“If this thing continues, then he should go visit a doctor”*

### Theme three: barriers to help-seeking behavior

The third general theme represented barriers to mental health help-seeking behaviors. This theme comprised of three sub-themes including social stigma, self-stigma, and masculinity.

#### Social stigma

Social stigma accounted for participants beliefs regarding how they would be perceived by others and society if they were to seek help for a mental health concern. Participants were most concerned with perceptions stemming from family and friends. The stigma associated with mental health may not only influence a person’s beliefs and attitudes towards them, but can also influence their behaviors (e.g., being out casted, losing friends). Participants frequently used terms such as “crazy” and “normal” when describing someone with or without a mental illness, respectively. Examples of participant quotes representative of this subtheme include the following:

*“Stigma. … If people figure out you're talking to someone … then you get the crazy label.”*

*“Oh, and fear of being outed, I guess, *per se*. Like if your friends find out that you're going to counseling they're like, what's wrong with you?”*

*“The stigma associated with them if someone found out like, ‘Oh you went to a mental health place. So you must be crazy or you're not stable. Don't talk to me. I need friends who are normal.’”*

#### Self-stigma

Another barrier identified was the self-stigma associated with a mental illness. Self-stigma captured how participants would view themselves should they receive help for a mental health issue. Participants questioned whether a mental health diagnosis made them unstable or out-of-control of their life. Analysis of this subtheme presented in multiple ways including perceptions of vulnerability. Examples of participant quotes representative of this subtheme include the following:

*“The biggest mental block would simply be going there [to get help] admits you have a problem. That you don't have control of what you're doing. That's a scary thing to admit.”*

*“I feel like it's probably kind of scary … ‘Maybe I'm like having a mental illness. … Does that make me stupid? Does that make me dangerous? Does that make me this or that?’”*

*“Pride. … you don't want to be known as one of the people who has to go get help. You want to feel like you can work things out yourself.”*

#### Masculinity

The third subtheme within the barriers theme was masculinity. This subtheme accounted for participants’ expressed beliefs regarding how males “should function in society.” Analyses revealed that traditional gender norms were a salient concern when males considered seeking mental health services and that they were a major obstacle to help-seeking. Most participants discussed males needing to be perceived as strong by hiding their feelings and by figuring out their own problems. Admitting a problem, not being able to solve one’s own problems, and even talking about one’s feelings meant that males were not manly and thus were weak and socially undesirable. Males’ perception was that females do not have the same societal pressures placed on them pertaining to mental health help-seeking. Examples of participant quotes representative of this subtheme include the following:

*“There's a stigma against men going to get mental healthcare, because it's perceived as some kind of weakness. It's expected for women to be, say, open with their feelings. With men, it's the complete opposite.”*

*“Especially among men, it's considered a weakness in a lot of groups of people (pause) it's not masculine to receive help.”*

*“Society tells us we have to be manly and just hold it in, and you can't be talking about your emotions. You're supposed to be the man of the house, the man of the family. You're not supposed to show any sign of weakness.”*

*“There's a lot of societal pressure … that males don't really have to seek help, you just figure it out on your own.”*

## Discussion

Attending to mental health literacy among male college students is critical for improving student health, well-being, and success as males are more likely to adopt maladaptive coping behaviors as opposed to mental health help-seeking behaviors. The purpose of the current study was to address noted gaps in the literature by exploring the multiple facets of mental health literacy among male college students in the U.S. By means of qualitative methodology grounded in Jorm’s mental health literacy conceptual framework, the current study explored undergraduate males’ knowledge of signs and symptoms, attitudes, beliefs, and perceived susceptibility to depression, anxiety, and stress, in addition to recommended help-seeking behaviors and barriers to care. The current study revealed three general themes including (a) knowledge of signs and symptoms (physiological, behavioral, and emotional); (b) recommended intra- and interpersonal help-seeking; and (c) barriers to help-seeking behaviors. Individually, these themes present with enlightening sub-themes; yet, when triangulated, findings present an interesting triadic interplay that provides insight for practice translations. Each theme will be discussed individually followed by a discussion regarding the triadic interplay between themes and sub-themes.

First, the identification with physiologic and behavioral signs and symptoms of anxiety, depression, and stress as opposed to emotional provides insight as to key indicators of poor mental health from the college male perspective. Participants expressed the familiarity of physical issues such as headaches, digestive issues, and rapid heart rate, in addition to behaviors such as inability to sleep, eat, irritability (i.e., yes, this happens to me). On the other hand, participants expressed an unfamiliarity to anxiety, depression, and stress as a mental health issue (i.e., no, this has not happened to me). However, although these physiologic and behavioral indicators were identified in the presenting vignettes, many were perceived as “normal” to the college experience. This was especially true for diagnoses that seemed common to the college experience, such as stress and anxiety. This is troublesome as identification of symptoms is a predictor of help-seeking [20].

Second, despite knowledge of signs and symptoms of mental health issues, participants varied on recommended care strategies based on (a) proximity to self and (b) the perceived mental health issue. For example, the current study reveals that the further removed the male is to himself, the more likely they were to recommend seeking professional services. College males are more likely to recommend to themselves “do nothing” and “wait it out”, as compared to recommending a medical provider for their friend, and a mental health provider to a stranger. This is supported by the findings of Rickwood and colleagues [19] where participants were more likely to recommend services to others than to use them themselves. College males in the current study revealed that talking to their male friend about physical and behavioral issues and advising him to seek a medical provider is much easier and less worrisome than discussing mental health. This finding also brings to light the importance of involving the university medical health services in the screening of mental health issues.

Further, results of the current study reveal that seeking professional mental health services for themselves was only expressed when presented with the depression vignette. These results support the work of others in that males tend to seek care only when in crisis [4]. Alternatively, as described previously, male college students are more likely to engage in unhealthy coping mechanisms in place of seeking care [4, 12, 14]. Subsequently, the adopting of maladaptive coping strategies may increase the likelihood of potential conduct violations. Similar to university student health services, university behavioral intervention teams and conduct offices should consider mental health screening when addressing student alcohol, drug, and behavioral conduct issues.

Third, the current study points to the importance of stigma and masculine motifs as barriers to help-seeking behaviors in addition to interpersonal help-seeking. A recent study by DeBate et al. [45] supports this point in that they found stigma negatively mediates the relationships between knowledge, motivation, and help-seeking among male college students. The work of Chuick and colleagues [15] and Oliffe and colleagues [38] support this idea by indicating that traditional ideas of masculinity negatively impact males help-seeking behavior in a cyclical fashion. Decreasing the role of masculinity and stigma in dictating males experience with mental health is paramount to successful programs; however, it is difficult to parse the exact effect of both.

**Fig. 1:**
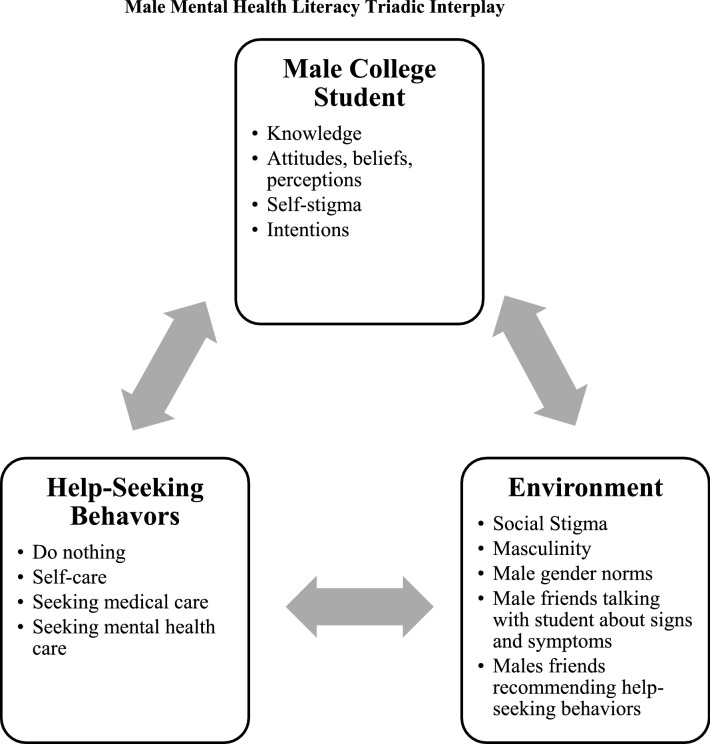
Male mental health literacy triadic interplay

Finally, interpreting these themes independently should be done with caution as the current study reveals an interesting triadic interplay between the person (i.e., male college student), their help-seeking behavior, and their environment. As depicted in Fig. 1, these three themes all influence each other; whereas, improving mental well-being among college males cannot be achieved by solely focusing and attending to one or two of these components. For example, masculine norms influence what males identify as indicators of poor health, in addition to influencing their intention to seek care. The choice of help-seeking behavior is influenced by self-stigma and social stigma. This also hold true for recommendations for help-seeking for their male friends. Engaging in self-care or “doing nothing” reinforces the masculine norms and self-stigma. The triadic interplay creates a persistent pattern that may be considered too difficult to break.

This study sought to better understand the factors impacting mental health literacy among male undergraduate college students and the analysis provides a detailed description of their knowledge, perception towards help-seeking, and barriers. As with most qualitative studies, the generalizability of this study is limited, and the findings cannot be extended beyond this population (e.g., females, males not attending university, males residing outside of the U.S.). This has been well-documented in qualitative research, thus generalizability beyond this population is not the purpose of the current study [64]. For example, the population was comprised of males who have access to resources due to being students, location can only be generalized to local university, and the vignettes were only specific to depression, anxiety, suicide ideation, and stress. Further, although the authors took note in data collection via a trained male interviewer, due to the level of trust and comfort in discussing mental health, there may have been socially desirable responses as well as self-censorship. Second, considerations of study results should note limitations presented by the vignettes. As development of the vignettes employed DSM-5 criteria as a guide, they should not be interpreted as representative of an individual diagnosed with generalized anxiety disorder nor depression. This may have limited the ability for participants to correctly identify representative mental health issues. To that end, translation of DSM-5 criteria for generalized anxiety disorder may have provided a better reflection of how these disorders manifest in a U.S. population. Nonetheless, despite certain limitations, the current study adds to the current literature regarding mental health literacy among college males in addition to translation to practice in institutions of higher education.

Despite limitations, the current qualitative study adds additional literature regarding college male mental health literacy. Moreover, a triadic interplay (person, environment, and behavior) may be noted to provide avenues for improving mental health literacy among male college students. In addition to addressing mental health literacy among male college students, perhaps improvements could be made by improving mental health literacy of college student healthcare providers. By re-perceiving this triadic interplay from an asset-based approach instead of a deficit-based approach, implications for practice can be made. For example, social marketing programs could be developed that are grounded in positive masculinity and provide action items to confidential on-line therapy assessments, modules, and programs such as TAO (Therapy Assistance Online [65]). Another recommendation is to develop paradigms for mental health around the physiological and behavioral signs of poor mental health. Mental health literacy training programs could be implemented for student health providers whereas they can be identified as first-line providers to secondary prevention of mental health issues. Future research should be implemented to further explore the dynamic relationships between these concepts in addition to development and evaluation of interventions aimed at improving mental health literacy and help-seeking behaviors among male college students.

## Data Availability

The datasets generated during and/or analyzed for the current study are not publicly available due to IRB approval criteria but are available from the corresponding author on reasonable request.
